# Supplementary material for: Multi-focused image fusion algorithm based on multi-scale hybrid attention residual network
Source: PLoS One. 2024 May 24;19(5):e0302545. doi: 10.1371/journal.pone.0302545 (PMC11125476; doi:10.1371/journal.pone.0302545)
Supplement: Attachment — Submitted filename: Response to Reviewers.pdf3.23.pdf [file pone.0302545.s001.pdf]

Original Manuscript ID: PONE-D-23-44021

Original Article Title: "Multi-focused image fusion algorithm based on multi-scale hybrid attention residual network"

To: Editor-in-Chief

Re: Response to reviewers

Dear Editor,

Thank you for allowing a resubmission of our manuscript, with an opportunity to address the reviewers' comments.

We are uploading (a) our point-by-point response to the comments (below) (response to reviewers), and (c) a clean updated manuscript (*Main Manuscript).*

Best regards,

<Liu> et al.

Reviewer#1, Concern # 1: How to weight the two different components in the loss function needs to be further explained and demonstrated.

Author response: Thanks for your suggestion, we changed the loss function in line 360-374 of the article.

Reviewer#1, Concern # 2: The evaluations of Tables 2 and 3 need more explanations especially on these quantitative paramters demonstrated.

Author response: Many thanks for your suggestion and sorry for our carelessness, we explained these quantitative parameters on lines 497-499 and 508-510 in the article.

Reviewer#2, Concern # 1: The content of this paper is incomplete, all figures are not provided.

Author response: Thank you for your suggestion. I am sorry for our carelessly. We have carefully checked the article and made up the missing figures, such as lines 338-340 in the article.

Reviewer#2, Concern # 2: Please cite relevant works from the past 3 years in the literature section.

Author response: Thank you for your valuable comments. We have added relevant works in the past three years to references 3-7 and references 46-51.

Reviewer#2, Concern # 3: in formulas 3 and 4, the symbols used differ from those appearing in the formulas, for example, φCAM and φ CAM. It is suggested to change for consistency to facilitate readers understanding.

Author response: Thank you for your comments. Our φ_{cfm} in Equation 3 represents the channel feature map, and φ_{CAM} in Equation 4 represents the output of the channel attention module.

Reviewer#2, Concern # 4: Why didn't the symbol ϵ(·) below formula 4 and the symbol φ below formula 10 appear in both formulas?

Author response: Thank you for your suggestion, sorry for our carelessness, $\varepsilon(\cdot)$ in Eq. 4 should be $\varepsilon(\cdot)$, we wrote it wrong and we modified it in line 291. ϕ in Eq. 10 is redundant and should be deleted. We have made changes and deletions in the original manuscript.

Reviewer#2, Concern # 5: What does S' mean in formula 10? Please provide an explanation below formula 10 for better understanding by the readers.

Author response: Thank you for your comments. S' represents the fused image features, we add it on line 349 of the article.

Reviewer#2, Concern # 6: Is Lsimm in contribution 3 the same as Ls in formula 13? If not, please provide clarification.

Author response: Thank you very much for your suggestion, I apologize for our carelessness, the Lsimm in contribution 3 is identical to the Ls in Eq. 13, We make a modification in Eq. 13.

Reviewer#2, Concern # 7: Eight methods were proposed in the comparative analysis, but only seven are presented in Table 1. Is the GF-IMF method not included for comparison?

Author response: Thank you for your suggestion and sorry for our carelessness. In the comparative analysis, it should be compared with seven methods, and the GF-IMF method is not included. We deleted it in line 428 of the article.

Reviewer#2, Concern # 8: The ablation study is insufficient. The authors only provide the ablation experiment of Loss function.

Author response:. Thank you very much for your suggestion, we have added the ablation experiment of the module in line 486 in the article.

Reviewer#2, Concern # 9: All parameters in formulas should be described and explained.

Author response: Thanks for your suggestion, we checked the parameters in the article and added unexplained parameters, such as lines 338-340 of the article.

Reviewer#2, Concern # 10: Lp and Lsimm are the classical loss function. In view of this point, the contribution 3 can not be regarded as the innovation.

Author response: Thank you very much for your comments, we have made new improvements to the loss function on lines 360-374 of the article.

Reviewer#3, Concern # 1: The paper claims robust performance across various datasets but lacks rigorous testing on diverse, real-world scenarios. The primary reliance on the MS-COCO2012 dataset raises concerns about the model's ability to generalize to different types of multi-focus images, especially in more complex or less controlled environments.

Author response: Thank you very much for your valuable advice. The images in the MS-COCO2012 dataset we used contain natural images and common pictures in life. The image fusion task on COCO dataset is difficult because of the complex background, the large number of targets, and the large number of scene categories. However, the fusion ability of our algorithm performs well on this dataset, and has better fusion ability than comparison algorithms. We can see that our trained model will generalize a bit better than the average model.

Reviewer#3, Concern # 2: While the paper introduces MSHRN, it does not sufficiently differentiate this approach from existing methods. The innovation in combining multi-scale features and attention mechanisms needs to be better highlighted, or the incremental nature of this improvement should be more transparent.

Author response: Thank you very much for your valuable advice. Based on your comments, we add the superiority of our method over existing methods in lines 245-276 of the introduction section of the article. Then, specific content is added in lines 350-358 of the multi-scale feature fusion module to make the innovation of the method more prominent.

Reviewer#3, Concern # 3: The role and effectiveness of the Up-and-Down Sampling Projection (UDP) module are not convincingly established. How does this module specifically contribute to the network's performance, especially in comparison to other edge-enhancement techniques?

Author response: Thank you very much for your valuable comments. We use Up and Down Sampled Projection Block (UDP) to collect high-frequency information to patch the edge of the extracted multi-scale

features, instead of using a dedicated method that consolidates feature representation and edge enhancement capabilities in a single network to patch the edge information of the image. We add lines 244 to 263 in the introduction section of the text for illustration.

Reviewer#3, Concern # 4: While the paper includes comparative analysis with several methods, the selection of these comparative algorithms appears limited. Inclusion of more recent and diverse state-of-the-art methods would better position the paper's contributions within the current research landscape.

Author response: Thank you very much for your valuable comments, and according to your suggestions, we added the state of the art method in line 432 of the article to compare with the algorithm in this paper.

Reviewer#3, Concern # 5: The reliance on subjective evaluation is a significant drawback. More rigorous quantitative metrics are needed to objectively assess the performance of the proposed method. Also, the manuscript does not provide a clear rationale for the choice of the specific metrics used.

Author response: Thanks for your comments, we have added more stringent evaluation metrics on lines 396-424 of the paper to objectively evaluate the performance of the proposed method. We elaborate and explain the specific evaluation metrics used.。

Reviewer#3, Concern # 6: The paper lacks detailed explanation and justification for the choice of network architecture, including the number of layers, filter sizes, and specific configurations of the MSHRN and UDP modules. Such details are crucial for reproducibility and critical assessment.

Author response: Thank you very much for your suggestions. Your suggestions are very important to us. We added the specific layers of the network architecture in the line 216-221 of the article, explained the size of the filter in the line 297-298, and elaborated the specific configuration of MSRB and UDP modules in the line 511-519 of the article.

Reviewer#3, Concern # 7: The combination of structural similarity function loss (Ls) and pixel function loss (Lp) is not novel. A more thorough justification for this specific combination and its parameter tuning is needed, including why this combination is more effective than others.

Author response: Thank you very much for your suggestions. We have further modified the loss function in lines 360-374 of the article.

Reviewer#3, Concern # 8: The paper does not provide sufficient theoretical grounding for the proposed method, particularly in how the MSHRN and UDP modules contribute to the overall performance improvement. More theoretical analysis or insights into why this architecture works effectively for multi-focus image fusion is needed.

Author response: Thank you very much for your suggestions. We provide more theoretical analysis on how MSRB and UDP modules can help improve the overall performance in lines 245-276 of the article, explaining why this architecture can be effectively used for multi-focus image fusion.

Reviewer#3, Concern # 9: The manuscript does not adequately address the computational efficiency of the proposed method, which is a crucial factor in real-world applications. Information about the model's computational complexity, memory requirements, and execution time would provide a more comprehensive assessment of its practical viability.

Author response: Thank you very much for your suggestions. We analyze and compare the complexity and computational efficiency of the model in line 520-525 of the article.
